# Regulation of visual Wulst cell responsiveness by imprinting causes stimulus-specific activation of rostral cells

**DOI:** 10.1038/srep42927

**Published:** 2017-02-23

**Authors:** Tomoharu Nakamori, Tomomi Kato, Hiroyuki Sakagami, Kohichi Tanaka, Hiroko Ohki-Hamazaki

**Affiliations:** 1 College of Liberal Arts and Sciences, Kitasato University, Sagamihara, Kanagawa 252-0373, Japan; 2 Department of Anatomy, Kitasato University School of Medicine, Sagamihara, Kanagawa 252-0373, Japan; 3 Laboratory of Molecular Neuroscience, Medical Research Institute, Tokyo Medical and Dental University, Bunkyo-ku, Tokyo 113-8510, Japan; 4 The Center for Brain Integration Research, Tokyo Medical and Dental University, Bunkyo-ku, Tokyo 113-8510, Japan

## Abstract

Imprinting behaviour in chicks can be induced exclusively during a short period after hatching. During this period, visual information on the imprinting stimulus is conveyed to the visual Wulst (VW) in the telencephalon, which corresponds to the visual cortex of mammals, and then to the memory-storing region known as the intermediate medial mesopallium. These two regions are indispensable for imprinting. We previously showed that imprinting training altered the response pattern of the VW to the imprinting stimulus; however, the precise distribution of cells and the mechanism involved with this altered response remains unclear. Here we showed that a specific population of rostral VW cells responded to the imprinting stimulus by analysing the subcellular localization of *Arc/arg3.1* transcripts in VW cells. GABAergic parvalbumin (PV) cells are abundant in the dorsal region of this area, and imprinting training doubled the number of activated PV-positive neurons. An injection of bicuculline, a GABA(A) receptor antagonist, in the dorsal VW disturbed the rostral distribution of responsive cells and thus resulted in a lack of imprinting. These results suggest that activated PV cells restrict VW cells response to dorsal area to form a specific imprinting pathway.

Newly hatched chicks of precocial birds learn the characteristics of an object to which they are exposed and acquire a preference for that object. This phenomenon, called imprinting^1^, is a specific form of learning which occurs only during a short period early in development and by which the robust and long-lasting memory is formed.

Taking advantage of imprinting in the domestic chicks in the studies of learning of memory, neural mechanisms of imprinting have been extensively studied^2^,^3^. A telencephalic region called intermediate medial mesopallium (IMM) which corresponds to the mammalian association cortex was the first to be found as a region involved in imprinting^4^. And then, another telencephalic region, the visual Wulst (VW) which corresponds to the mammalian visual cortex was also found to be indispensable for visual imprinting in chicks^5^. We previously identified a neural pathway connecting the VW and IMM that is of crucial importance for visual imprinting^2^. Visual information processed by the retina is conveyed to the dorsolateral anterior thalamic nucleus and then transferred to the telencephalon in the interstitial nucleus of the hyperpallium apicale (IHA) layer, which is situated in the dorsal VW^5^. In the VW, the information is then transmitted to a more ventral region, including the hyperpallium intercalatum (HI) and hyperpallium densocellulare (HD) layers, and the processed information converges onto the core nucleus of the HD (HDCo) before arriving at the IMM^5^. Using *in vivo* imaging of neuronal activity, we demonstrated that the imprinting training altered regions of the VW that respond to visual stimuli^6^, suggesting that information relevant to the imprinting stimulus is processed in the VW. We postulated that this processing triggered an enhanced response of HDCo cells to the imprinting stimulus^7^. Additional analyses targeting the VW are needed to address the cellular and molecular mechanisms of the plastic changes involved in this process.

An immediate-early gene known as activity-regulated cytoskeleton-associated protein (*Arc*)^8^ or activity-regulated gene (*Arg3.1*)^9^ is rapidly expressed after neural activation. Several studies showed that changes in *Arc* gene expression are driven by learning in both rodents^10^,^11^,^12^,^13^ and birds^14^,^15^. After synaptic activation, the *Arc* transcripts shift from the nucleus to the cytoplasm within 15 min in rodents^16^. Through detection of the subcellular localization of *Arc* transcripts by fluorescence *in situ* hybridization (FISH), the activation history of neurons during two temporally distinct events could be determined^10^. A correlation between the strength or establishment of imprinting and the expression of the immediate-early gene product cFos in the VW was reported, suggesting that the expression pattern of the *Arc* gene can be used to detect activated and inactivated cells^5^,^17^,^18^. In the present study, we completed a spatial analysis of the subcellular localization of *Arc* transcripts using FISH. We showed that imprinting training altered the spatial distribution of neurons in the HI/HD layers of the VW that were responsive to the imprinting stimulus. We used this method again and determined that parvalbumin (PV)-positive inhibitory neurons in the IHA showed plastic changes and thus may have an important role in this process. Imprinting training activated a subset of PV cells that appear to facilitate the specification and enhancement of the rostral VW cells crucial for eliciting imprinting behaviour.

## Results

### Temporal changes in the intracellular localization of *Arc* transcripts after image presentation

**Figure 1:**
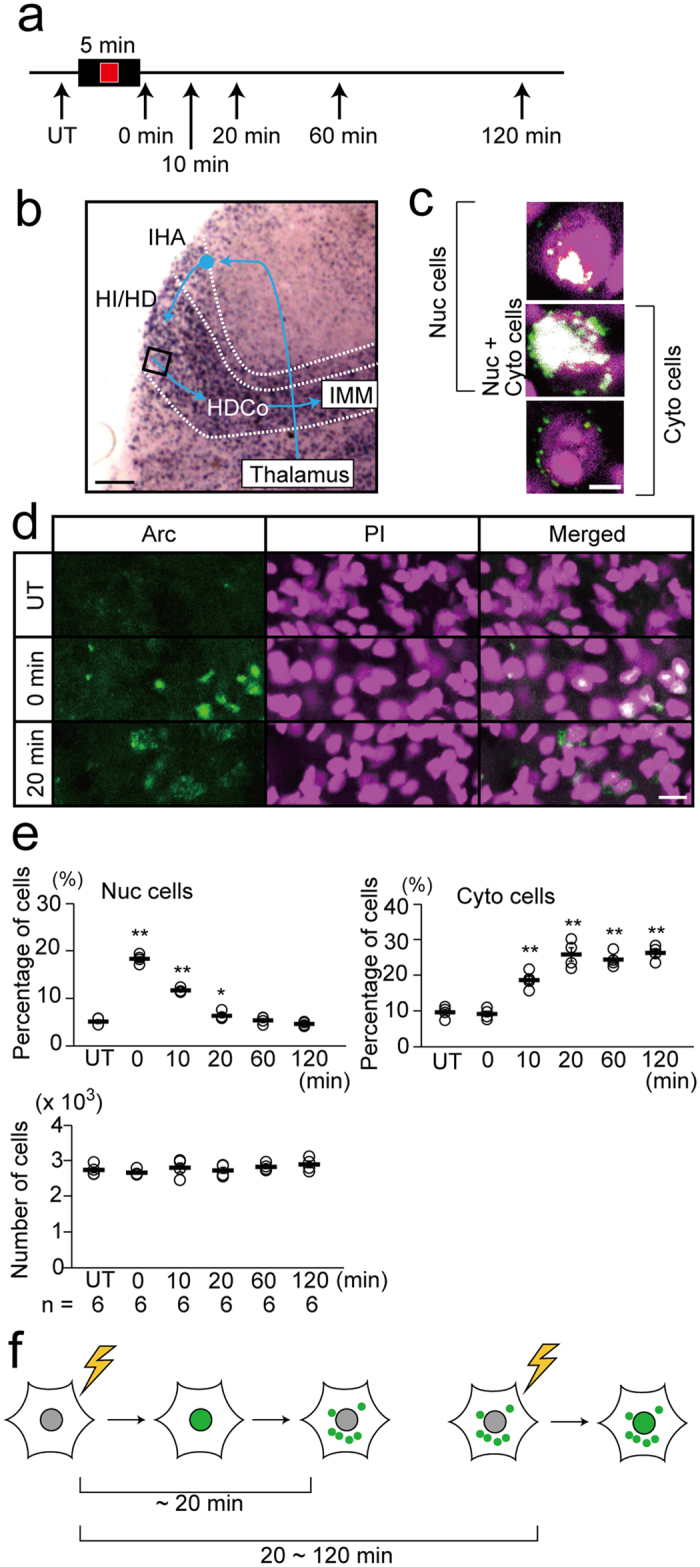
*Arc* was expressed rapidly in the nucleus after 5-min image presentation, and translocated to the cytoplasm in 10–20 min. (**a**) Experimental design. At P1, Chicks were individually put into the apparatus, exposed for 5 min to a moving red square presented on the monitor, returned to the dark incubator (except for 0 min condition) and VW cells were analysed at indicated time points. Chicks kept in the dark incubator (untreated; UT) were used as controls. (**b**) The expression pattern of *Arc* transcripts in the VW and schema showing the pathway for visual imprinting in the chick telencephalon. The square area shows regions analysed by FISH. (**c**) Representative images of cells with nuclear transcripts alone (upper), with both nuclear and cytoplasmic transcripts (middle; Nuc + Cyto cells), and with cytoplasmic transcripts alone (bottom). The upper and middle cells are Nuc cells, and the middle and bottom cells are Cyto cells. (**d**) *Arc* transcripts (green) in UT, at 0 min, and 20 min. Nuclei are stained with propidium iodide (PI, magenta). Nuclear and cytoplasmic localizations of *Arc* transcripts are prominent at 0 min and 20 min, respectively. (**e**) Temporal changes in the percentage of cells with nuclear transcripts (Nuc cells; upper left) and with cytoplasmic transcripts (Cyto cells; upper right). The percentage of Nuc cells was significantly high at 0 min and 10 min after presentation, and the percentage of Cyto cells increased at 10–120 min after presentation of the image. The number of analysed cells was not significantly different among conditions (bottom). (**f**) Schema indicating translocation of *Arc* transcripts with time. *Arc* is expressed in the nucleus immediately after the 5-min image presentation, and most of the transcripts translocate to the cytoplasm within 20 min after image presentation. Consequently, when the 2nd presentation is performed within 20–120 min after the first one, cells with both nuclear and cytoplasmic transcripts are regarded as the cells that responded to both stimuli. *p < 0.05; **p < 0.01. Scale bar = 250 μm (**b**), 5 μm (**c**), and 20 μm (**d**).

We first examined time-dependent changes in the subcellular localization of *Arc* transcripts in chick VW cells after image presentation (Fig. 1a). In the defined area of the VW (Fig. 1b), we calculated the proportion of cells with *Arc* transcripts in the nucleus (Nuc cells; Fig. 1c top, middle) and cytoplasm (Cyto cells; Fig. 1c middle, bottom). The cells with nuclear transcripts constituted 5% of the total cells in untreated chicks. This percentage increased to 19% immediately after the 5-min image presentation, remained significantly high (12%) after 10 min (Fig. 1d,e; untreated vs. 0 or 10 min, p < 0.01, t-tests), and then returned to baseline. The cells with cytoplasmic transcripts constituted 10% of the total cells in untreated chicks. This percentage increased to 19% 10 min after image presentation, reached 27% after 20 min and remained high until 120 min (Fig. 1d,e; untreated vs. 10, 20, 60 or 120 min, p < 0.01, t-tests). The number of cells observed was not significantly different among conditions (Fig. 1e bottom; × 10^3^; untreated, 2.72 ± 0.08; 0 min, 2.68 ± 0.07; 10 min, 2.75 ± 0.11; 20 min, 2.70 ± 0.07; 60 min, 2.75 ± 0.07; 120 min, 2.84 ± 0.09; untreated vs. 0 min, p = 0.45; vs. 10 min, p = 0.78; vs. 20 min, p = 0.81; vs. 60 min, p = 0.72; vs. 120 min, p = 0.21; t-tests). These results indicate that a 5-min image presentation immediately induced *Arc* transcripts in the nucleus of VW cells in chicks, mRNA began translocation to the cytoplasm approximately 10 min later, and transcripts were predominantly located in the cytoplasm after 20 min. Therefore, our analysis of the subcellular localization of *Arc* transcripts can reveal the activation history of VW cells after two sessions of image presentation separated by 20–120 min (Fig. 1f).

### Identification of cells that respond to two images presented 60 min apart

**Figure 2:**
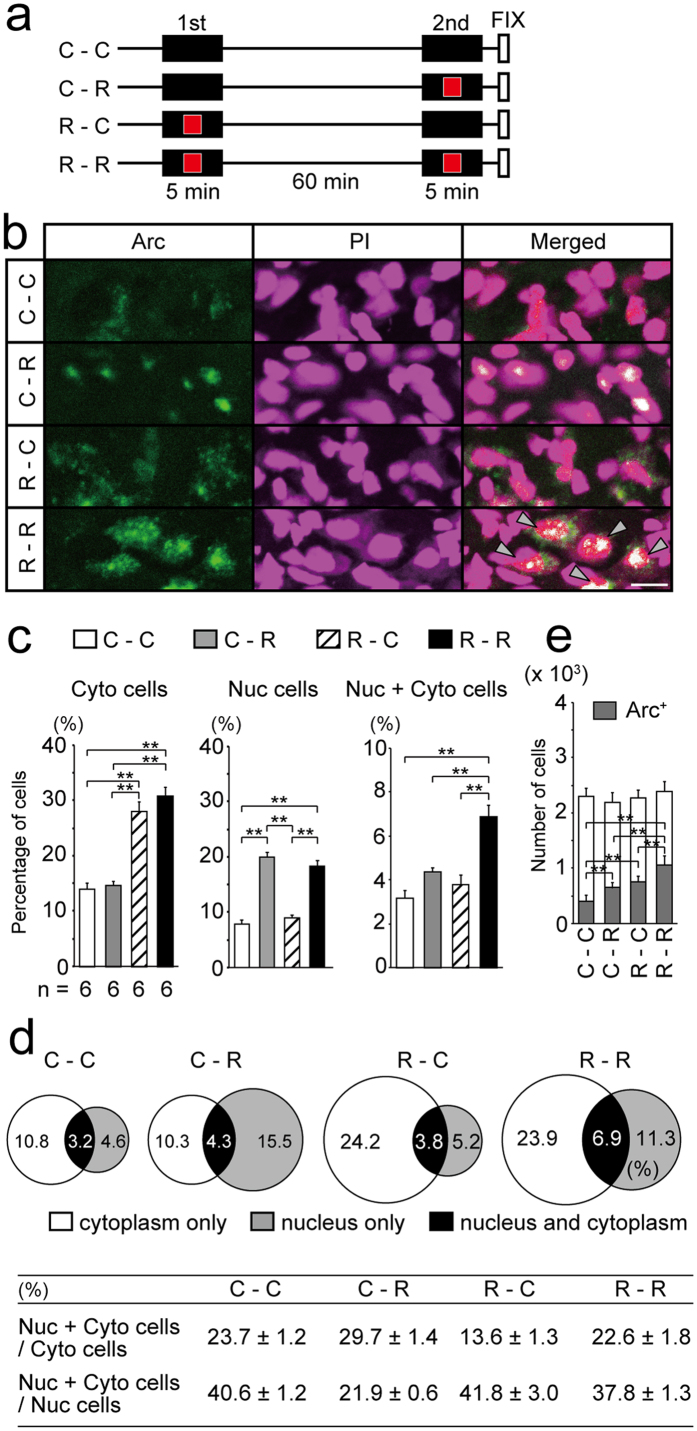
FISH analysis enables to detect responses of cells against two images presented with 60 min interval. (**a**) Experimental design. Chicks were placed in the apparatus for 5 min (1st session), returned to the dark incubator, and after 60 min, they were placed in the apparatus again for 5 min (2nd session). C, exposed to the black screen without any image; R, exposed to the moving red square presented on a black screen. (**b**) Representative images of *Arc* expression (green) in the VW of chicks in the indicated conditions. Nuclei are stained (PI, magenta). Gray arrowheads indicate the cells with both nuclear and cytoplasmic transcripts (Nuc + Cyto cells). (**c**) Note that the percentage of Cyto cells is significantly higher in chicks that were exposed to the moving red square during the 1st session compared to other chicks, and the percentage of Nuc cells is significantly higher in chicks that were exposed to the moving red square during the 2nd session compared to other chicks. The percentage of Nuc + Cyto cells in R–R chicks is significantly higher than that of other chicks. (**d**) Venn diagrams showing percentages of cells classified according to the *Arc* expressing pattern. The number is the mean value of 6 samples and the circle size correlates with the magnitude of number. Table shows the percentages of Nuc + Cyto cells to Cyto cells or Nuc cells in each condition. Mean ± SE calculated from 6 samples is shown. (**e**) Number of cells (gray plus white bar) was not significantly different among groups, but the number of *Arc* expressing cells differed between groups. **p < 0.01. Scale bar = 20 μm.

To validate the use of this analysis in our experimental design, we attempted to identify cells activated by images presented during the experimental sessions (Fig. 2a). FISH was performed on brains collected immediately after the second session, and we analysed the same region of the VW shown in Fig. 1b. The percentage of cells with cytoplasmic transcripts (Fig. 2b,c left) was higher in chicks exposed to the red square during the first session (R–C, 28%; R–R, 31%) than those exposed to the black control screen during the first session (C–C, 14%; C–R, 15%; F_(3, 20)_ = 41.26, p < 0.01, ANOVA; R–C vs. C–C or C–R and R–R vs. C–C or C–R, p < 0.01, post-hoc tests). Additionally, the percentage of cells with nuclear transcripts (Fig. 2b,c middle) was higher in chicks exposed to the red square during the second session (C–R, 20%; R–R, 18%) than those exposed to the black control screen during the second session (C–C, 8%: R–C, 9%; F_(3, 20)_ = 60.46, p < 0.01, ANOVA; C–R vs. C–C or R–C and R–R vs. C–C or R–C, p < 0.01, post-hoc tests). These results show that 20–30% of cells were responsive to the newly presented image, including approximately 10% that may respond non-specifically. The percentage of cells with both nuclear and cytoplasmic transcripts (Nuc + Cyto cells; Fig. 2b,c right) was higher in chicks exposed twice to the red square (R–R, 7%) than in those that were only exposed once (C–R, 4%; R–C, 4%) or not at all (C–C, 3%; F_(3, 20)_ = 18.18, p < 0.01, ANOVA; R–R vs. C–C, C–R, or R–C, p < 0.01, post-hoc tests). These results indicate that in R-R conditions, as shown in Fig. 2d, 31% of the total cells were activated during the first presentation (Cyto cells), only 7% of the total cells were re-activated during the second presentation of the same image (Nuc + Cyto cells), and 11% of the total cells were newly activated during the second presentation (cells with nuclear transcripts alone). The total number of cells observed was not significantly different among groups (Fig. 2e; ×10^3^; C–C, 2.32 ± 0.16; C–R, 2.23 ± 0.19; R–C, 2.29 ± 0.16; R–R, 2.40 ± 0.19; F_(3, 20)_ = 0.45, p = 0.72, ANOVA), whereas the number of *Arc*-positive cells was significantly different between groups (Fig. 2e; ×10^3^; C–C, 0.43 ± 0.09; C–R, 0.62 ± 0.08; R–C, 0.71 ± 0.09; R–R, 1.00 ± 0.19; F_(3, 20)_ = 55.81, p < 0.01, ANOVA; C–C vs. C–R, R–C or R–R and R–R vs. C–R or R–C, p < 0.01, post-hoc tests). These results show that this experimental protocol can identify the response of each cell against two images presented 60 min apart.

### Imprinting altered the number and distribution of responsive cells in the HI/HD

**Figure 3:**
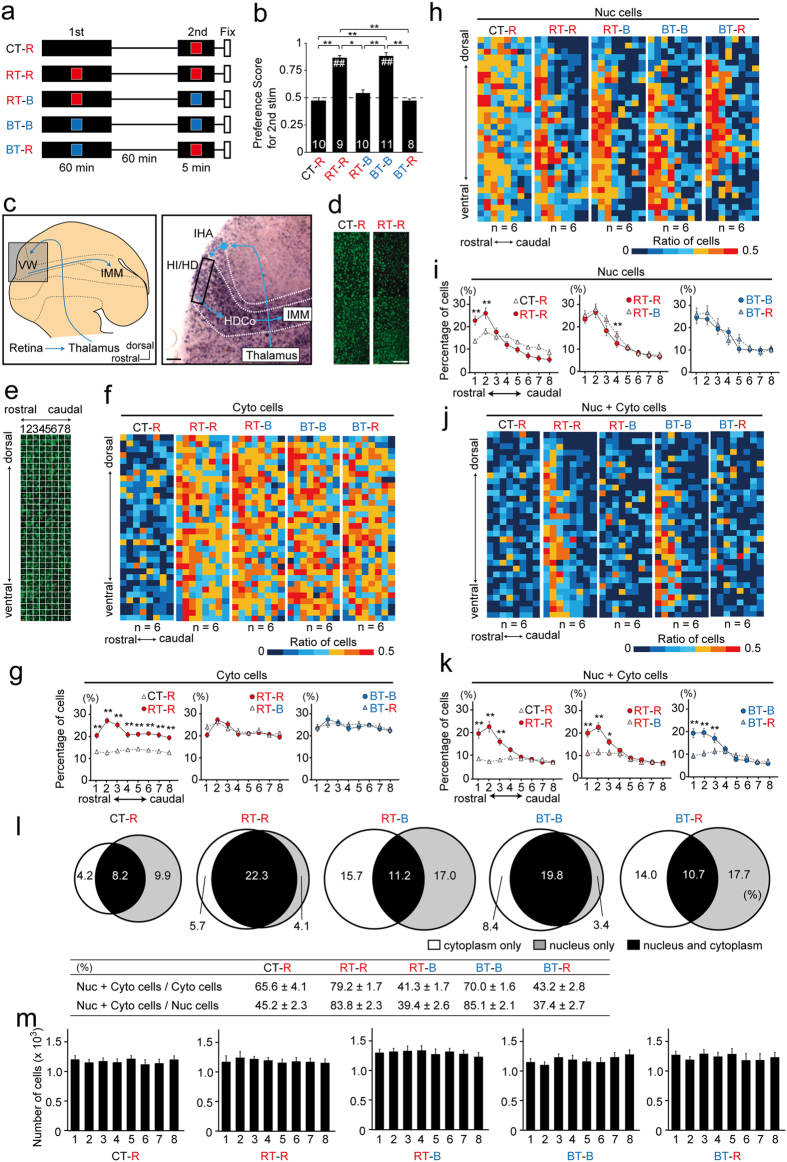
Visual imprinting localized the population of cells responsive to the imprinting stimulus. (**a**) Experimental design. Chicks were subjected to two sessions of exposure. During the 1st session, chicks were exposed to the black monitor without any image (CT), the moving red square (RT), or the moving blue square (BT). During the 2nd session, chicks were exposed to the moving red (R) or blue square (B). (**b**) RT–R and BT–B chicks showed significant preference to the 2nd stimulus. Number of chicks used are indicated in each bar. (**c**). Schema showing the pathway for visual imprinting in the chick telencephalon (left) and *Arc* expression in the enclosed region of the left schema (right). This photograph is identical to the one presented in Fig. 1b. The rectangular area shows analysed region. (**d**) An example of *Arc* expression of CT–R and RT–R chicks in the analysed regions. (**e**) Schema of the analysed region that was divided into 8 rostral-caudal × 32 dorsal-ventral areas. The percentages of Cyto cells, Nuc cells, and Nuc + Cyto cells to the total number of cells were calculated in each small area. (**f**) Heat maps were created from the mean value of the calculated percentages of Cyto cells. (**g**) Percentages of Cyto cells in each of the 8 rostral-caudal rectangular areas are plotted. Numerous Cyto cells are broadly distributed in each condition, except for in CT–R chicks. (**h**) Heat maps of Nuc cells. (**i**) Nuc cells are gathered in the rostral area in each condition, except for in CT–R chicks. (**j**) Heat maps of Nuc + Cyto cells. (**k**) In RT–R and BT–B chicks, Nuc + Cyto cells are gathered in the rostral area. (**l**) Venn diagrams and table created similarly as Fig. 2d, for the position 2. (**m**) Number of cells in the 8 rostral-caudal rectangular areas is shown for each condition. No significant differences were found among positions. *p < 0.05; **and ^##^p < 0.01. Scale bar = 250 μm (**c**) and 100 μm (**d**).

Next, we studied the distribution of cells in the HI/HD layers that responded to the imprinting stimulus or a new stimulus. Chicks were divided into 5 experimental groups according to the presented image (Fig. 3a). Sixty minutes of exposure to a moving red or blue square was shown to induce imprinting behaviour to the presented image^6^,^7^. As expected, RT–R and BT–B chicks during the second session showed response to the presented image with a preference score (PS) of 0.84 ± 0.04 and 0.86 ± 0.05, respectively, whereas CT–R, RT–B and BT–R chicks did not show this response with a PS of 0.46 ± 0.04, 0.56 ± 0.05 and 0.46 ± 0.03, respectively (Fig. 3b; RT–R or BT–B vs. 0.5, p < 0.01, respectively; CT–R vs. 0.5, p = 0.52; RT–B vs. 0.5, p = 0.41; BT–R vs. 0.5, p = 0.39; t-tests; F_(4, 43)_ = 19.21, p < 0.01, ANOVA; RT–R vs. CT–R or BT–R and BT–B vs. CT–R, RT–B or BT–R, p < 0.01; RT–R vs. RT–B, p < 0.05; post-hoc tests).

We performed FISH in a rectangular region in the most rostral part of the HI/HD layers (Fig. 3c–e) to compare the distribution of cells with *Arc* transcripts between CT–R and RT–R, RT–R and RT–B and BT–B and BT–R. We selected the first 6 chicks for each condition when arranged in chronological order of the behavioural experiments. The PSs of selected CT–R, RT–R, RT–B, BT–B and BT–R chicks were 0.48 ± 0.06, 0.83 ± 0.06, 0.51 ± 0.08, 0.87 ± 0.02 and 0.45 ± 0.05, respectively (see Supplementary Fig. S1; RT–R or BT–B vs. 0.5, p < 0.01; CT–R, RT–B or BT–R vs. 0.5, p > 0.3; CT–R vs. RT–R and BT–B vs. BT–R, p < 0.01; RT–R vs. RT–B, p < 0.05; t-tests). A substantial number of cells with cytoplasmic transcripts showed a broad distribution in RT–R, RT–B, BT–B and BT–R chicks but not CT–R chicks, which indicates that the imprinting stimulus presented to naïve chicks, evoked the response of a large number of cells in this area (Fig. 3f,g). In contrast, cells with nuclear transcripts in RT–R chicks were rostrally concentrated (Fig. 3h,i), and the percentage of cells with nuclear transcripts in the rostral area in RT–R chicks (Fig. 3i left, position 2; 26.4 ± 2.3%) was higher than CT–R chicks (18.1 ± 1.3%; RT–R vs. CT–R at position 2, see Supplementary Table S1 for ANOVA; p < 0.01, post-hoc tests). This rostral distribution was also observed for cells with both nuclear and cytoplasmic transcripts in RT–R chicks. The percentage of these cells in the rostral area was higher in RT–R chicks (Fig. 3j,k left, position 2; 22.3 ± 2.4%) than CT–R chicks (8.2 ± 0.8%; RT–R vs. CT–R at position 2, see Supplementary Table S1 for ANOVA; p < 0.01, post-hoc tests). As shown in Fig. 3l, at position 2 of the RT-R chicks 28% of the total cells responded during the first presentation, 22% of the total cells were re-activated during the second presentation of the same image, and only 4% of the total cells were newly activated during the second session. Among the cells that responded to the presented image during the first presentation, 79% (Nuc + Cyto cells/Cyto cells) also reacted to the same stimulus during the second presentation. Among the cells that responded to the image during the second presentation, 84% (Nuc + Cyto cells/Nuc cells) also reacted during the first presentation of the same image. A localised distribution of cells with nuclear transcripts and cells with both nuclear and cytoplasmic transcripts in RT–R chicks indicated that the imprinting training restricted the responding cells to the most rostral area of the VW.

To examine whether the results presented above depended on a specific imprinting stimulus, we compared the FISH results among RT–R, RT–B, BT–B and BT–R chicks. Cells with nuclear transcripts were distributed in the rostral region of the VW (Fig. 3h,i), indicating that the imprinting training rostrally restrained the responsive area. However, differences in the distribution of cells with both nuclear and cytoplasmic transcripts were prominent among these groups (Fig. 3j,k). The percentages of these cells in the rostral area were higher in RT–R chicks (Fig. 3k middle, position 2; 22.3 ± 2.4%) than RT–B chicks (11.2 ± 1.1%; RT–R vs. RT–B, position 2, see Supplementary Table S1 for ANOVA; p < 0.01, post-hoc tests). Similarly, the percentages of these cells in the rostral area were higher in BT–B chicks (Fig. 3k right, position 2; 19.8 ± 1.2%) than BT–R chicks (10.7 ± 0.9%; BT–B vs. BT–R, position 2, see Supplementary Table S1 for ANOVA; p < 0.01, post-hoc tests). In both RT–R and BT–B chicks, the percentage of cells with both nuclear and cytoplasmic transcripts was lower than cells with nuclear transcripts (Fig. 3l; RT–R, 22% vs. 26%; BT–B, 20% vs. 23%), whereas their distribution patterns were similar. Indeed, both the percentages of Nuc + Cyto cells to Cyto cells and Nuc + Cyto cells to Nuc cells exceeded 70% in these chicks (Fig. 3l). These results indicate that the majority of cells responsive to the imprinting stimulus after the establishment of imprinting were already activated during training (i.e. during the first session). Conversely, in RT–B and BT–R chicks, the distribution of cells with both nuclear and cytoplasmic transcripts was sparse and did not coincide with cells with nuclear transcripts (Fig. 3l; Nuc + Cyto cells/Cyto cells and Nuc + Cyto cells/Nuc cells, 37–43%). This indicates that the imprinting training fixed the reactive cells against the imprinting stimulus, and after the establishment of imprinting, most of the cells that reacted to the new stimulus in the second session were those that were not activated during the first session. For each condition, the number of cells was not significantly different among the 8 positions (Fig. 3m).

### Imprinting induced a population of parvalbumin-positive cells specifically responsive to the imprinting stimulus

**Figure 4:**
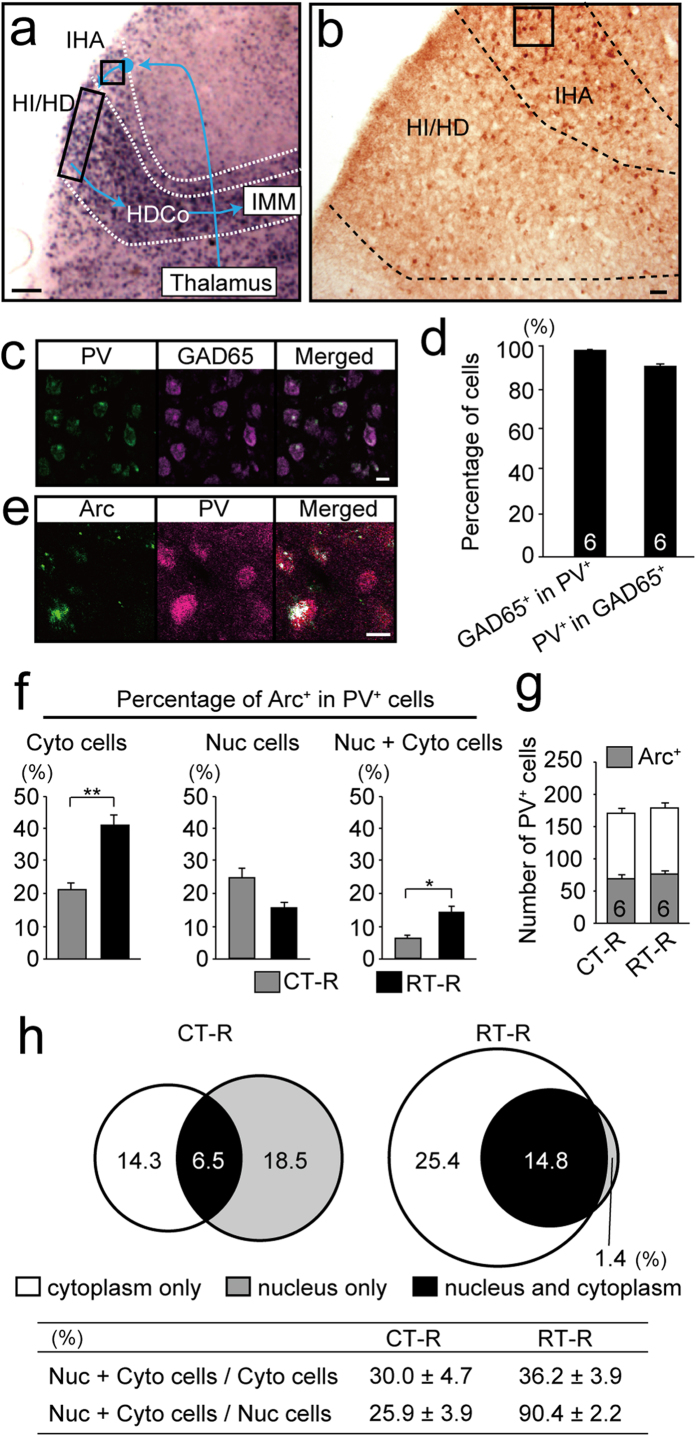
Imprinting altered activity of the parvalbumin-positive inhibitory neurons in the IHA, afferent to the HI/HD layers. (**a**) *Arc* Expression and the pathway for visual imprinting in the chick telencephalon. This schema is derived from the same photograph as those presented in Figs 1b and 3c. A black square in the IHA layer encloses the analysed region. (**b**) Image of immunostaining with anti-parvalbumin (PV) antibody. PV cells are prominent in the IHA layer. The enclosed area indicates the analysed region, which is identical to the region shown in a. (**c**). Fluorescent staining of PV protein (green) and GAD65 mRNA (magenta). (**d**) Almost all PV cells (98 ± 0.2%) express GAD65, and almost all GAD65-positive cells (91.0 ± 0.9%) express PV. (**e**) Fluorescent staining images of *Arc* transcripts (green) and PV protein (magenta) in RT–R chicks. (**f**) The percentages of Cyto cells, Nuc cells, and Nuc + Cyto cells among PV cells in RT–R and CT–R chicks were calculated. The percentages of PV-positive Cyto cells and Nuc + Cyto cells in RT–R chicks are higher than those in CT–R chicks. (**g**) Number of PV cells as well as *Arc*-positive PV cells in CT–R chicks was not significantly different from those of RT–R chicks. (**h**) Venn diagrams and table created similarly as Fig. 2d. *p < 0.05; **p < 0.01. Scale bar = 250 μm (**a**), 100 μm (**b**), 20 μm (**c**,**e**).

The IHA layer, which is dorsal to the HD layer (Fig. 4a), contains neurons that receive inputs from the dorsolateral anterior nucleus of the thalamus, a nucleus that relays visual information^19^,^20^. As we previously showed^18^, PV-expressing cells are abundant in the IHA layer (Fig. 4b). Most PV cells (98.0 ± 0.2%) in the IHA layer expressed the GABAergic neuronal marker GAD65, and most GAD65-positive cells (91.0 ± 0.9%) expressed PV (Fig. 4c,d). In RT–R chicks, *Arc* expression was induced in a subpopulation of the PV cells in the IHA (Fig. 4e). We examined if imprinting training altered the activity of PV neurons, which may influence the response of HI/HD cells to the imprinting stimulus. We performed a FISH analysis on PV cells in the IHA layer and compared the results of RT–R and CT–R chicks using the same samples as in Fig. 3 (Fig. 4f). Among PV positive cells in the IHA, the percentages of cells with cytoplasmic transcripts and cells with both nuclear and cytoplasmic transcripts in RT–R chicks (Cyto cells, 40.2 ± 2.4%; Nuc + Cyto cells, 14.8 ± 2.2%) were higher than those in CT–R chicks (Cyto cells, 20.8 ± 1.3%; Nuc + Cyto cells, 6.5 ± 1.4%; p < 0.01 and p < 0.05, respectively, t-tests). In contrast, the percentage of PV positive cells with nuclear transcripts in RT–R chicks (16.2 ± 2.1%) was lower than that in CT–R chicks, but this difference did not reach statistical significance (25.1 ± 3.5%, p = 0.06, t-test). The numbers of PV cells and *Arc*-positive PV cells in the IHA layer of CT–R (PV cells, 171.3 ± 6.7; *Arc*-positive PV cells, 68.1 ± 4.9) and RT–R chicks (PV cells, 178.7 ± 5.9; *Arc*-positive PV cells, 74.0 ± 4.6) were not significantly different (Fig. 4g; PV cells, p = 0.58; *Arc*-positive PV cells, p = 0.47; t-tests). These results indicate that in RT-R chicks, 40% of PV-positive cells in the IHA were activated during training, including approximately 15% that showed a specific response to the imprinting stimulus during the second session; newly activated cells during the second session were almost absent (Fig. 4h). In sharp contrast, in CT-R chicks 21% of PV-positive cells were activated during training, including only 7% that were re-activated; 19% were newly activated during the second session.

### Inhibition of GABAergic synaptic transmission in the IHA disrupted imprinting behaviour and the localization of cells responsive to the imprinting stimulus

**Figure 5:**
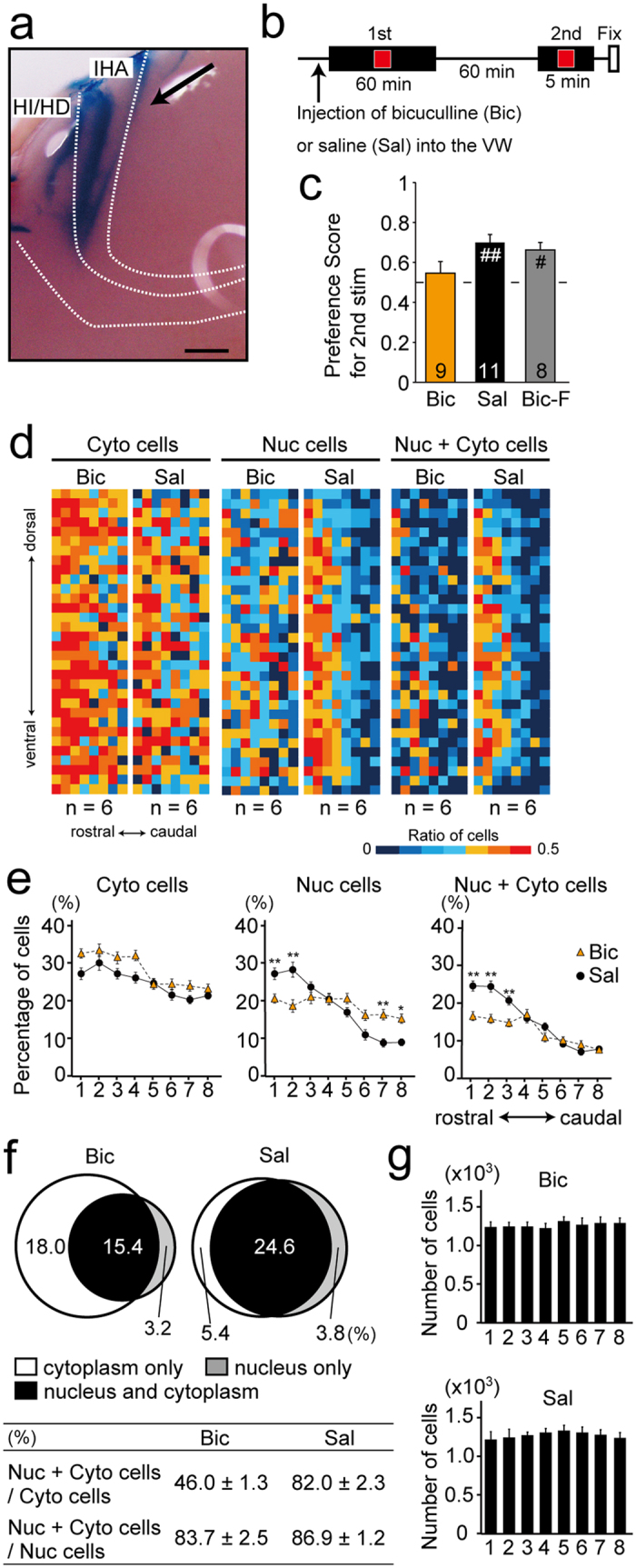
Blockade of inhibitory transmission disrupted the localization of cells responsive to the imprinting stimulus and imprinting behaviour. (**a**) Solution containing 0.1% Evans blue dye was injected in the IHA layer; the injected site is recognized by blue staining.(**b**) An antagonist of GABA (A) receptors, bicuculline (Bic), or saline (Sal) was injected into the IHA, 5 min before the 60-min presentation of the moving red square (1st session). After a 60-min rest, the moving red square was presented again for 5 min (2nd session). (**c**) Bic injected chicks did not show preference for the imprinting stimulus during the 2nd session. Bic-F, bicuculline was injected outside the VW by failure. Number of chicks used are indicated in each bar. (**d**) Heat maps showing the percentages of Cyto cells, Nuc cells, and Nuc + Cyto cells in Bic or Sal treated chicks in the rectangular region in the HI/HD layers shown in Fig. 4a. (**e**) In Bic treated chicks, Cyto cells are more numerous and more broadly distributed, and Nuc cells and Nuc + Cyto cells are more uniformly distributed than in Sal treated chicks. (**f**) Venn diagrams and table created similarly as Fig. 2d, for the position 2. (**g**) Cell number was not significantly different among positions in Bic or Sal treated chicks. *and ^#^p < 0.05; **and ^##^p < 0.01. Scale bar = 250 μm.

GAD-positive PV cells were dominant in the IHA (Fig. 4b–d); therefore, we assumed that these cells may inhibit the activity of excitatory neurons in the IHA. To examine if this function of PV neurons in the IHA is required for imprinting behaviour and the modification of HI/HD cell activity, we blocked the activity of A-type GABA receptors in the IHA. Microinjections into the IHA layer before training (Fig. 5a,b) revealed that the A-type GABA receptor antagonist bicuculline (Bic) but not saline (Sal) reduced preference score to a level not significantly different from the chance level of 0.5 (Fig. 5c; Bic, 0.54 ± 0.07; Sal, 0.70 ± 0.06; Bic vs. 0.5, p = 0.60; Sal vs. 0.5, p < 0.01; t-tests). Chicks in which bicuculline was injected outside of the VW due to technical error (Bic–F; see Supplementary Fig. S2) showed the response to the imprinting stimulus (Bic–F, 0.68 ± 0.06; vs. 0.5, p < 0.05; t-test). For the FISH analysis, we selected the first 6 chicks when arranged in chronological order of the behavioural experiments with the bicuculline and saline injected groups. The PSs were 0.54 ± 0.09 for bicuculline injected chicks and 0.74 ± 0.06 for saline injected chicks (see Supplementary Fig. S3; Bic vs. 0.5, p = 0.70; Sal vs. 0.5, p < 0.05; t-tests). The results of the FISH analysis showed that the percentages of cells with cytoplasmic transcripts in the bicuculline-treated chicks were higher than those in the saline-treated chicks (Fig. 5d left, 5e left; main effect of condition F(1,10) = 12.62, p < 0.01; ANOVA). Although cells with nuclear transcripts in the saline-treated chicks were concentrated in the rostral area, these cells in bicuculline-treated chicks were broadly distributed (Fig. 5d middle, 5e middle, position 2; Bic, 18.5 ± 1.6%; Sal, 28.4 ± 1.5%; see Table S1 for ANOVA; p < 0.01, post-hoc tests). Similarly, cells with both nuclear and cytoplasmic transcripts in the saline-treated chicks were concentrated more rostrally in the HI/HD layers; however, this distribution pattern was not evident in the bicuculline-treated chicks (Fig. 5d right, 5e right, position 2; Bic, 15.4 ± 1.1%; Sal, 24.6 ± 1.2%; see Table S1 for ANOVA; p < 0.01, post-hoc tests). Differences in the distribution of cells with nuclear transcripts or cells with both nuclear and cytoplasmic transcripts between the bicuculline- and saline-treated chicks (Fig. 5d–f) are reminiscent of the differences between CT-R and RT-R chicks (Fig. 3h,i left, 3j,k left). The number of cells among the 8 positions was not significantly different in bicuculline- and saline-treated chicks (Fig. 5g). This result indicates that the blockade of GABAergic transmission in the IHA affected the response pattern of HI/HD cells and led to a failure in imprinting. Thus, a subset of PV cells in the IHA may inhibit excitatory neurons in this layer; furthermore, the activation of these PV cells by the imprinting stimulus during training was required for both the altered response pattern of HI/HD cells and the establishment of imprinting.

## Discussion

In the present study, we examined the response of cells to an imprinting stimulus by analysing the subcellular localization of *Arc* transcripts in VW cells. First, we showed that the temporal changes in the intracellular localization of *Arc* transcripts successfully detected VW cells that were activated by two image presentations with a 60-min interval. We found that after imprinting training, rostrally distributed cells in the HI/HD layers became responsive to the imprinting stimulus but not a new stimulus. Next, we extended the analysis to the IHA layer, which contains abundant GABAergic PV-positive cells. Upon imprinting training, a subset of these cells became specifically activated by the imprinting stimulus. Inhibition of A-type GABA receptor activity in the IHA resulted in the suppression of imprinting, and the rostral concentration of responsive cells was no longer observed.

We previously showed that in visual imprinting, information is processed in the VW and transmitted to the IMM^2^. Furthermore, we reported that neurons in the HDCo of the VW, which send long axons to the nucleus afferent to the IMM, are activated by imprinting training^7^. However, how visual information on an imprinting stimulus is processed in the VW before it is conveyed to the HDCo cells remained unclear. In this study, we found that the altered response of VW cells to the imprinting stimulus was induced during or immediately after imprinting training and was crucial for the development of imprinting behaviour. Furthermore, the altered response in the VW resulted in the localization of responsive cells, which supports our previous findings obtained using intrinsic optical recording of neuronal activity in the VW^6^. When comparing the results of R-R and RT-R chicks, the ratios of Nuc + Cyto cells to Cyto cells or Nuc cells are higher in RT-R chicks than R-R chicks (Figs 2d and 3l). This result suggests that the imprinting training fixed a substantial number of responding cells. In RT–R and BT–B chicks, the distribution pattern of cells with nuclear transcripts roughly overlapped with cells with both nuclear and cytoplasmic transcripts. In RT–B and BT–R chicks, numerous cells with nuclear transcripts were distributed in the rostral area, whereas cells with both nuclear and cytoplasmic transcripts were sparsely distributed (Fig. 3). These results indicated that after imprinting training, only a small number of cells, if any, responded to both the imprinting stimulus and a new stimulus; furthermore, a different population of cells became responsive to either the imprinting stimulus or the new stimulus. We speculate that the neurons responsive to the imprinting stimulus, which may be fixed by imprinting training, are excitatory neurons and send information relevant to the imprinting stimulus to HDCo cells. Consequently, these HDCo cells may be activated and a positive feedback mechanism mediated by NR2B-containing NMDAR^7^ induces long-term potentiation and imprinting. Future studies examining the efferent connections of the activated cells in the HI/HD regions are needed to clarify this point.

The role of inhibitory neurons, including PV cells, on neural plastic changes in infancy was studied in the context of rodent sensory experiences. The regulation of the critical period and progression of ocular dominance plasticity in the visual cortex are attributed to the maturation and activity of PV cells^21^,^22^,^23^. Transplantation of embryonic inhibitory neurons obtained from the medial ganglionic eminence reinstates ocular dominance plasticity in young mice that are past the age of the critical period and adult mice, thus highlighting the importance of inhibitory neurons in plasticity^24^,^25^.

Concerning imprinting behaviour in chicks, GABA- or PV-positive cells in the IMM are activated during learning^26^. However, the detailed mechanisms of the neural modifications mediated by inhibitory neurons during early learning are largely unknown^3^. We previously showed that surgical ablation of VW or chemical deletion of HDCo cells inhibited imprinting behaviour, but did not affect general vision and visual acuity^5^,^6^. In the present study, we speculated that the inhibitory signals originating from PV cells are critical for the modification of the local neural network, which is involved in successful visual imprinting. Our results indicated that PV cells located in the IHA layer exert their effects on excitatory neurons in the same layer that projects to neurons in the HI/HD layer. Consequently, the response of these HI/HD neurons to the imprinting stimulus is stabilized. However, the involvement of PV cells that directly project to neurons in the HI/HD layers cannot be excluded. A detailed morphological analysis of these PV cells is needed to clarify this point. Regardless, PV cells in the IHA layer may contribute to the formation of the neural pathway critical for imprinting.

## Materials and Methods

### Animals

Fertilized eggs from White Leghorn chickens (*Gallus gallus domesticus*) were obtained from local suppliers (Akebono Farm, Hiroshima, Japan, and Nihon Layer, Gifu, Japan) and were allowed to develop in an incubator at 37.7 °C with moderate moisture and in quasi-constant darkness. After hatching, chicks of either sex were kept in groups in the same incubator. The present study was carried out in accordance with the Guidelines for the Treatment of Experimental Animals of Tokyo Medical and Dental University and with Regulations for the Care and Use of Laboratory Animals in Kitasato University. The experimental protocols described in this paper were approved by the Institutional Animal Care and Use Committee for Tokyo Medical and Dental University, and for Kitasato University.

### Imprinting device

Visual imprinting device has been described previously^5^,^6^,^7^,^18^. Briefly, a running wheel connected to a custom-made computer system was used to record the chick’s movements toward or away from the display (Muromachi Kikai, Tokyo, Japan). A 15-inch liquid crystal monitor was placed on each side of the wheel, and an image was displayed on one of the two monitors with black background (only for the experiments described in Fig. 1, S1503-T, EIZO, Ishikawa, Japan; for others, Flex Scan L367, Nanao, Ishikawa, Japan). The images were generated by a visual stimulus generator system (VSG; Cambridge Research Systems, Ltd., Kent, UK). The square had 8.6-cm-long sides (24° of the visual field) and bounced left and right horizontally on the screen at a rate of 7.3 cm/s (20.9°/s). In the experiments, all colors had a luminance of 10.36 cd/m^2^, and the Commission Internationale de l’Eclairage *xy* chromaticity coordinates of the tested colors were as follows: red with S1503-T monitor, 0.635 and 0.330 (for only the experiments described in Fig. 1); red with Flex Scan L367 monitor, 0.658 and 0.307 (for other experiments); blue with Flex Scan L367 monitor, 0.15 and 0.062 (for all experiments).

### Presentation of visual stimulus and evaluation of visual imprinting

Chicks at 24–48 h after hatching (P1) were individually placed in the running wheel. For the experiments described in Fig. 1, chicks were exposed to a moving red square on the screen for 5 min, returned to the dark incubator (except for those analysed immediately) and their brains were analysed at various time points after image presentation. Chicks kept in the dark incubator (untreated; UT) were also used. For the experiments other than those described in Fig. 1, chicks were exposed to the red or blue square presented on the monitor, or the black monitor without any image, for 5 min (only for experiments described in Fig. 2) or 60 min (first session). We have already confirmed that the 60-min but not the 5-min image presentation could induce the imprinting behaviour^7^. Then chicks were placed back into the same incubator as before the exposure. After 60-min resting in the incubator, they were placed into the imprinting apparatus again and exposed to the image or the black screen for 5 min (second session). The direction and number of wheel revolutions induced by chick were recorded. We calculated the preference score (PS) as described previously^6^,^27^ using the following formula as an index of success of visual imprinting: PS = The number of wheel revolutions toward the display during 5-min second session/The total number of wheel revolutions during 5-min second session. Because exposure to one more stimulus during second session inevitably affects *Arc* expression, we did not measure the PS for another stimulus.

### General histological methods

Whole brains were fixed with 4% paraformaldehyde for about 16 h at 4˚C, cryoprotected by immersing in 30% sucrose for 48 h, embedded in the Tissue-Tek O.T.C. Compound (Sakura Finetek Japan, Tokyo, Japan) and frozen in powdered dry ice. Using a cryostat (CM1900 or CM3050S, Leica Microsystems, Wetzlar, Germany), 30-μm-thick sagittal sections were prepared. The sections were mounted on the slide glasses, dried, and kept at −80 °C.

### Fluorescence *In situ* hybridization (FISH) and immunostaining

Chick *Arc/Arg3.1* gene fragment (AJ272062.1; nt 96–1337) was amplified using chick brain cDNA by PCR with following primers: forward 5′- TCA TGC AGC TGG ACA ATG TCA CCA -3′ and reverse 5′- AGA TTG CCG TCT CCA TCC AGC TAA C -3′. The amplified fragment was cloned into the pBSIISK (−) vector (Stratagene, La Jolla, CA, USA). The clone for chick glutamic acid decarboxylase 65 (GAD65) gene was described previously^18^. Gene-specific sense and antisense digoxigenin (DIG)-labeled cRNA probes were generated using a Roche RNA labeling kit (Roche Applied Science, Indianapolis, IN, USA). *In situ* hybridization (FISH) was performed as described previously^18^ with modifications.

The sections mounted on the slide glasses were washed twice with 0.01 M phosphate buffer (PB), treated with proteinase K (1 μg/ml) in Tris-EDTA buffer (pH 8.0) at 37 °C for 10 min, and acetylated with 0.25% acetic anhydride in 0.1 M triethanolamine for 10 min. Hybridization was performed at 60 °C for 14–16 h in a solution containing 50% formamide, 2% blocking reagent (Roche Applied Science), 5 × saline sodium citrate (5 × SSC; 0.75 M NaCl, 0.075 M Trisodium citrate dehydrate), 0.1% N-lauroylsarcosine, 0.1% sodium lauryl sulfate, and DIG-labeled cRNA probe. The sections were washed sequentially at 60 °C with a solution containing 50% formamide and 1 × SSC for 30 min, with 1 × SSC for 30 min, and with 0.1 × SSC for 30 min, and then treated with DIG-1 buffer (0.1 M Tris-HCl pH7.5, 0.15 M NaCl, 0.1% Tween 20). After quenching in PB containing 0.3% H_2_O_2_ for 30 min, they were incubated in blocking buffer (5% bovine serum albumin, 5% normal goat serum in DIG-1 buffer) for 1 h at room temperature. After blocking, sections were incubated for 30 min at room temperature with horseradish peroxidase-conjugated anti-DIG antibody (1:1000, Roche Applied Science) in blocking buffer. After washing with DIG-1 buffer, using TSA plus DNP system (PerkinElmer, MA, USA), the sections were treated with DNP amplification reagent for 15 min. The sections were washed in DIG-1 buffer, and were treated with Alexa 488-conjugated anti-DNP antibody (1:500, Thermo Fisher Scientific, IL, USA). No specific signal was observed when the sections were processed with DIG-labeled sense RNA probes. The cell nucleus was stained with propidium iodide (PI; 1:1000, Thermo Fisher Scientific).

For immunohistochemistry, sections were treated as described^5^,^18^,^27^ and mouse anti-parvalbumin antibody (1:1000, Santa Cruz, CA, USA) was applied to the sections for 12 h at 4 °C. After washing with 0.01 M phosphate buffered saline (PBS) containing 0.5% triton X-100, the sections were reacted with a polymer reagent including peroxidase and goat anti-mouse IgG antibody (Dako Envision kit/HRP; DakoCytomation, Glostrup, Denmark) for 1 h at room temperature. They were treated with 0.1% 3, 3′-diaminobenzidine to visualize peroxidase, and then washed with 0.01 M Tris-HCl buffer (pH 8.0) containing 1 mM EDTA to stop the reaction. Images were acquired under constant exposure condition using a Leica (DMRA) microscope equipped with a DFC300FX digital camera and Leica Application Suite software.

For the double fluorescence staining for either *Arc* mRNA or GAD65 mRNA and parvalbumin protein, the *in situ* hybridization was performed first, and then the immunohistochemistry was performed. Alexa 568-conjugated anti-mouse IgG antibody (1:1000, Sigma-Aldrich, MO, USA) was used as a secondary antibody for immunohistochemistry. The cell nucleus was stained with TO-PRO-3 iodide (1:1000, Thermo Fisher Scientific).

### Cell count

We used 6 chicks for the histological analysis for each condition and selected 3 sagittal sections per brain that included the VW. For each section, a 250 μm × 250 μm square area of the rostral-ventral part of the HD layer (Figs 1 and 2), a 250 μm × 1000 μm rectangular area of the HI/HD layers (Figs 3 and 5), or a 250 μm × 250 μm square area of the IHA layer (Fig. 4) of the VW was selected as a region of interest (ROI). Fluorescence images were acquired using a confocal laser microscope (LSM710; Carl Zeiss, Oberkochen, Germany). Images were taken from sections and without knowledge of the presented images or presentation protocols used, the number of cells with different subcellular localization of *Arc* transcripts (nuclear alone, both nuclear and cytoplasmic, or cytoplasmic alone) was counted with the aid of a software (NIH Image; National Institutes of health, MD, USA). For the rectangular ROI, the whole area was divided to 8 (rostral-caudal) × 32 (dorsal-ventral) small areas, and the counting was performed in each area. The whole number of cells was also counted, and the percentages of cells with *Arc* transcripts in the cytoplasm (Cyto cells), in the nucleus (Nuc cells), and in both the nucleus and cytoplasm (Nuc + Cyto cells), to the total number of cells were calculated. For the rectangular ROI, based on the calculated percentage, the heat map was created (Figs 3f,h,j and 5d). The percentage in each of the 8 rostral-caudal rectangular areas (position 1 to 8) was also calculated and plotted (Figs 3g,i,k and 5e). For the double-stained sections, we judged the single or double-positive cells by observation, and similarly counted the cell number.

To validate the judgement of subcellular localization of *Arc* transcripts, we compared the number of cells counted by two experimenters. The percentages of Nuc cells, Cyto cells, and Nuc + Cyto cells, calculated based on the counts by two experimenters were not significantly different, respectively (Nuc cells, 12.0 ± 0.7% vs 11.8 ± 0.7%, p = 0.81; Cyto cells, 19.6 ± 1.8% vs 18.6 ± 1.4%, p = 0.67; Nuc + Cyto cells, 8.8 ± 0.8% vs 8.5 ± 0.8%, p = 0.67; t-tests). And the differences of two calculated ratios were 5.5 ± 1.3% for Nuc cells, 6.4 ± 1.8% for Cyto cells, and 7.1 ± 0.9% for Nuc + Cyto cells.

### Microinjection into the VW

Microinjection was performed according to the procedures described previously^5^,^27^. A free-hand injection into the left VW (7 mm rostral from the bregma, 2 mm left from the midline and 1-mm depth from the skull surface) was performed to unesthetized P1 chicks. Either an antagonist of A-type GABA receptor, bicuculline (10 μM, 2 μl; Sigma-Aldrich) or saline (2 μl), containing 0.1% Evans blue dye was injected with a syringe (Hamilton Company, Reno, NV, USA).

### Statistical analysis

All data in this study are expressed as means ± SEM. The number of animals used is indicated in each figure or legend. A one-sample t-test was used to examine whether the PS values differed significantly from chance (0.5)^6^,^27^, indicated by # or ## in Figs 3b and 5c. After confirming equality of variances by F-test, the differences between two experimental groups were analysed using the Student’s t-test (Figs 1e and 4f,g). We used one-way ANOVA, followed by post-hoc Tukey-Kramer test to compare the values between conditions (Figs 2c,e,3b,m and 5g). A two-way ANOVA followed by post-hoc Tukey-Kramer test was used to compare the percentage of *Arc* expressing cells or number of cells in each of the 8 rostral-caudal regions (position 1–8) between two experimental groups (Figs 3g,i,k and 5e), and the results of two-way ANOVA were shown in Supplementary Table S1. Differences were regarded as statistically significant at p < 0.05.

## Additional Information

**How to cite this article**: Nakamori, T. *et al*. Regulation of visual Wulst cell responsiveness by imprinting causes stimulus-specific activation of rostral cells. *Sci. Rep.* **7**, 42927; doi: 10.1038/srep42927 (2017).

**Publisher's note:** Springer Nature remains neutral with regard to jurisdictional claims in published maps and institutional affiliations.

## Supplementary Material

Supplementary Information
