# Supplementary material for: Regulation of visual Wulst cell responsiveness by imprinting causes stimulus-specific activation of rostral cells
Source: Sci Rep. 2017 Feb 23;7:42927. doi: 10.1038/srep42927 (PMC5322328; doi:10.1038/srep42927)
Supplement: Supplementary Information [file srep42927-s1.pdf]

Supplementary Information

Regulation of visual Wulst cell responsiveness by imprinting causes stimulus-specific activation of rostral cells.

Tomoharu Nakamori, Tomomi Kato, Hiroyuki Sakagami, Kohichi Tanaka and Hiroko Ohki-Hamazaki

Supplementary Table S1. Results of two-way ANOVA

		main effect of condition	main effect of position	condition × position interaction
Fig. 3g (Cyto cells)	CT-R vs RT-R	F(1,10)=241, p=3E-08 **	F(7,70)=2.99, p=0.008 **	F(7,70)=2.658, p=0.017 *
	RT-R vs RT-B	F(1,10)=0.063, p=0.807	F(7,70)=4.341, p=5E-04 **	F(7,70)=0.43, p=0.88
	BT-B vs BT-R	F(1,10)=3E-06, p=0.999	F(7,70)=2.795, p=0.013 *	F(7,70)=0.845, p=0.554
Fig. 3i (Nuc cells)	CT-R vs RT-R	F(1,10)=2.457, p=0.148	F(7,70)=43.1, p=6E-23 **	F(7,70)=14.66, p=1E-11 **
	RT-R vs RT-B	F(1,10)=7.373, p=0.022 *	F(7,70)=106.5, p=1E-34 **	F(7,70)=2.324, p=0.034 *
	BT-B vs BT-R	F(1,10)=2.958, p=0.116	F(7,70)=52.56, p=2E-25 **	F(7,70)=2.786, p=0.013 *
Fig. 3k (Nuc + Cyto cells)	CT-R vs RT-R	F(1,10)=63.23, p=1E-05 **	F(7,70)=22.39, p=1E-15 **	F(7,70)=20.69, p=9E-15 **
	RT-R vs RT-B	F(1,10)=25.35, p=5E-04 **	F(7,70)=30.21, p=9E-19 **	F(7,70)=9.937, p=2E-08 **
	BT-B vs BT-R	F(1,10)=29.6, p=3E-04 **	F(7,70)=36.51, p=6E-21 **	F(7,70)=17.87, p=2E-13 **
Fig. 5e	Cyto cells	F(1,10)=12.62, p=0.005 **	F(7,70)=13.08, p=1E-10 **	F(7,70)=0.854, p=0.547
	Nuc cells	F(1,10)=0.458, p=0.514	F(7,70)=33.68, p=5E-20 **	F(7,70)=16.14, p=2E-12 **
	Nuc + Cyto cells	F(1,10)=23.14, p=7E-04 **	F(7,70)=59.62, p=5E-27 **	F(7,70)=10.63, p=5E-09 **

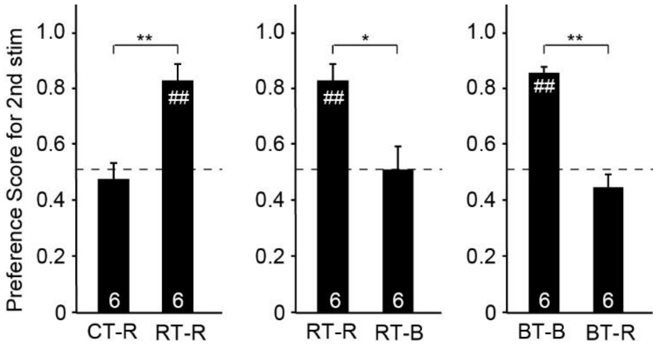

Supplementary Figure S1. Preference scores of chicks used for FISH analysis.

RT-R and BT-B chicks showed preference to the stimulus, but CT-R, RT-B and BT-R chicks did not. ##, $p<0.01$; one-sample t-tests. * and **, $p<0.05$ and 0.01, respectively; t-tests. n=6 for each groups.

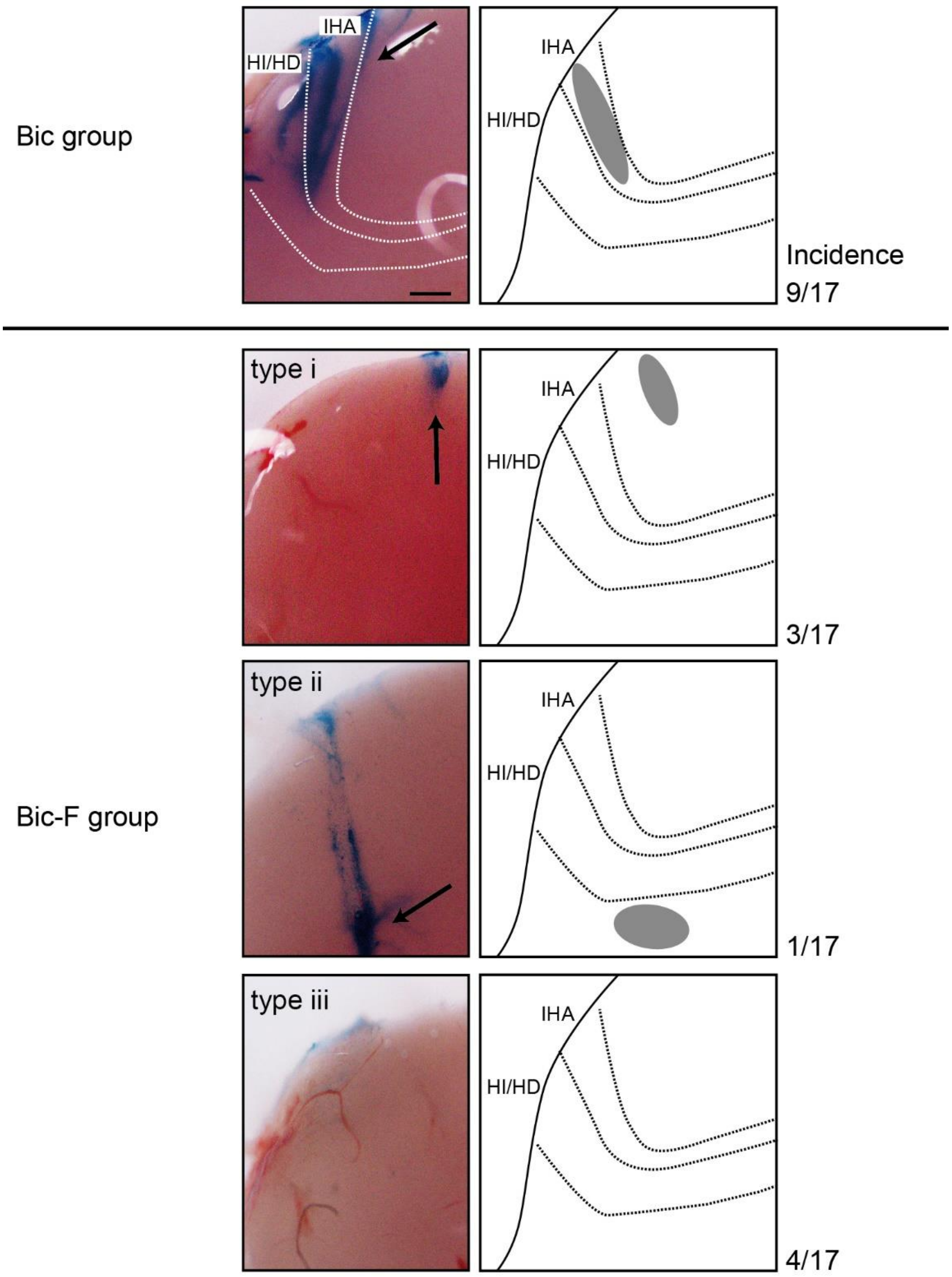

Supplementary Figure S2. Injected site for Bic and Bic-F group.

Injected site was judged by the staining caused by Evans blue added in the bicuculline (Bic) solution. Chicks in which staining was detected in the IHA layer were included in Bic group. In Bic-F groups, staining was detected dorsal to the IHA (type i), ventral to the VW (type ii), or not at all in the brain (type iii).

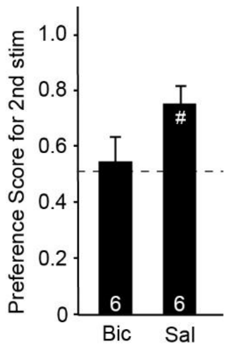

Supplementary Figure S3. Preference scores of chicks used for FISH analysis.
Saline injected chicks (Sal) showed preference to the stimulus, but bicuculline (Bic) injected chicks did not. #, $p<0.05$; one-sample t-tests. n=6 for each group.
